# Quebec Spinal Muscular Atrophy Newborn Screening Program: The First Year Experience

**DOI:** 10.3390/ijns11040089

**Published:** 2025-10-05

**Authors:** Emilie Groulx-Boivin, Ariane Belzile, Cam-Tu Émilie Nguyen, Amélie Gauthier, Nicolas Chrestian, Catherine Michaud-Gosselin, Yves Giguère, Marie-Thérèse Berthier, Jean-François Soucy, Anne-Marie Laberge, Maryam Oskoui

**Affiliations:** 1 Departments of Pediatrics and Neurology & Neurosurgery, Montreal Children’s Hospital, McGill University, Montreal, QC H4A 3H9, Canada; 2 Research Institute, McGill University Health Centre, Montreal, QC H4A 3J1, Canada; 3 Department of Neurosciences and Pediatrics, Division of Pediatric Neurology, Centre Hospitalier Universitaire Sainte-Justine, University of Montreal, Montreal, QC H3T 1C5, Canada; 4 Department of Pediatrics, Division of Pediatric Neurology, Centre Mère-Enfant Soleil, Laval University, Quebec City, QC G1V 0E8, Canada; 5 Department of Molecular Biology, Medical Biochemistry and Pathology, Faculty of Medicine, Laval University, Quebec City, QC G1V 0A6, Canada; 6 Newborn Screening of Quebec Province, Department of Medical Biology, CHU de Québec-Université Laval, Quebec City, QC G1V 4G2, Canada; 7 Department of Pediatrics, Division of Medical Genetics, Centre Hospitalier Universitaire Sainte-Justine, University of Montreal, Montreal, QC H3T 1C5, Canada; 8 OPTILAB Montréal—CHU Sainte-Justine, Centre Hospitalier Universitaire Sainte-Justine, University of Montreal, Montreal, QC H3T 1C5, Canada

**Keywords:** newborn screening, spinal muscular atrophy, pediatric, *SMN2*, genetic disorder, Canada

## Abstract

Clinical trials in spinal muscular atrophy (SMA) have shown that early treatment improves outcomes, prompting inclusion in newborn screening (NBS) programs worldwide. The province of Quebec launched its SMA NBS program in October 2023, with a rapidly progressive implementation. We describe the program’s first-year experience, focusing on screening yield, birth prevalence, clinical outcomes, and challenges. In the first year, 6 of 67,933 newborns screened positive for SMA, all subsequently confirmed by diagnostic testing. Of these, 4 newborns (67%) had two *SMN2* copies and 2 newborns (33%) had four copies. Additionally, one symptomatic compound heterozygote infant presented during this period, indicating a provincial birth prevalence of 1 in 9705 live births (95% CI: 1:20,032–1:4701). Two newborns with two *SMN2* copies were symptomatic at initial consultation; one transitioned to palliative care and died at 43 days of life. Surviving newborns initiated treatment at a median age of 30 days (range: 9–103 days), with four receiving onasemnogene abeparvovec and one nusinersen. Motor outcomes at three or six months were stable or improved among treated infants. Overall, the Quebec SMA NBS pilot program successfully identified affected newborns, facilitated early access to therapy, and provided the first provincial estimate of SMA birth prevalence. Improved sample shipping and processing times are needed to maximize the program’s impact, which is expected with full automation.

## 1. Introduction

Spinal muscular atrophy (SMA) is a severe neuromuscular disorder characterized by degeneration of anterior horn cells, leading to progressive muscle atrophy and weakness. It is caused by disruptions in the survival motor neuron 1 (*SMN1*) gene located on chromosome 5q, leading to a deficiency in the SMN protein, essential for motor neuron maintenance [1]. Approximately 95% of affected individuals have a homozygous absence of exon 7/8 on *SMN1*, while the remaining 5% are compound heterozygotes with absent exon 7/8 on one allele and a pathogenic variant in exon 7/8 on the other [2]. Disease severity correlates with the number of copies of the paralogous gene *SMN2*, with a lower copy number associated with earlier disease onset and a more severe phenotype [3].

Health Canada has approved three disease-modifying therapies for SMA: nusinersen (approved June 2017) [4], onasemnogene abeparvovec (December 2020) [5], and risdiplam (April 2021) [6]. Nusinersen is an intrathecally administered antisense oligonucleotide that modifies *SMN2* splicing and is indicated for all subtypes of 5q-SMA across all ages [4,7]. Onasemnogene abeparvovec is an intravenous viral-mediated *SMN1* gene replacement therapy, indicated for pediatric patients with 5q-SMA and up to three *SMN2* copies or infantile-onset SMA [5,7]. Risdiplam, an oral small molecule that also modifies *SMN2* splicing, is indicated for all subtypes of 5q-SMA across all ages [6,7].

Public reimbursement for these therapies exists across all Canadian provinces, although policies regarding treatment eligibility for newborns with no clinically evident signs vary. In Quebec, the Institut national d’excellence en santé et en services sociaux (National Institute for Excellence in Health and Social Services, INESSS) recommends public reimbursement of nusinersen for infants predicted to develop SMA types 1, 2, or 3, without specifying the number of *SMN2* copies. In contrast, reimbursement for onasemnogene abeparvovec is limited to infants with a confirmed genetic diagnosis of 5q-SMA and two or three *SMN2* copies. While risdiplam had been approved by Health Canada for use in infants under two months of age, it had not yet received a reimbursement recommendation for this age group during the study period in the province of Quebec.

Clinical trials across all three therapies have demonstrated improved outcomes when treatment is initiated early [8,9,10,11]. This evidence has led to the inclusion of SMA in an increasing number of newborn screening (NBS) programs worldwide, enabling earlier diagnosis and treatment initiation [12]. Following successful pilots in Ontario [13] and Alberta [14], the province of Quebec launched its SMA NBS program in October 2023, in an opt-in verbal consent framework, the same process as for the other conditions screened. This manuscript presents the first-year experience of the Quebec SMA NBS program, providing an overview of its progressive implementation, screening yield, clinical outcomes, and key programmatic challenges. It also offers the first estimate of SMA birth prevalence in Quebec.

## 2. Materials and Methods

All newborns born in Quebec are eligible for the provincial NBS program: the Programme québécois de dépistage néonatal sanguin (PQDNS) [15]. This program is offered free of charge, with explicit verbal consent obtained from families. Dried blood spot samples are typically collected within the first 24–48 h of life, either by nurses in hospital settings or by midwives in birthing centers or in the community. The same sample is used for SMA screening, biochemical testing, and all other conditions included in the NBS program. Historically, uptake has been high, with 97–98% of eligible families participating [16]. All samples are sent via Canada Post regular mail and analyzed at a unique fiduciary center, CHU de Québec—Université Laval, on a daily basis from Monday to Friday, excluding statutory holidays. The choice of hospital for referring newborns who test positive is up to the parents, taking into account their postal code and their affiliation with a region linked to a designated referral center for positive cases. These reference centers are pediatric neurology services designated by the Ministry of Health to manage the child’s care after a positive screening. Following referral and evaluation, a confirmatory test is performed on a clinical blood sample at the Centre Hospitalier Universitaire Sainte-Justine.

### 2.1. SMA NBS Program Description

The Quebec SMA NBS program was launched on 2 October 2023, initially screening newborns from the Centre Hospitalier Universitaire Sainte-Justine. Province-wide implementation was carried out progressively over two months, beginning 11 December 2023. During the study period, the automation and information technology components at the CHU de Quebec laboratory were still being implemented; therefore, the program had not yet reached its final operational phase.

As determined by the Ministry of Health, only the absence of exon 7 was verified. Two testing methods were applied to dried whole blood spots on filter paper to determine the number of copies of exon 7 on the *SMN1* gene and the number of copies of the *SMN2* gene. The first-tier assay was conducted using real-time PCR (QuantStudio DX, Thermo Fisher Scientific, Waltham, MA, USA) to detect the presence or absence of *SMN1* gene copies, with extraction and PCR carried out using the Revvity NeoMDX kit. Samples testing positive on the first tier underwent second-tier testing using droplet digital PCR (ddPCR, QX ONE, Bio-Rad Laboratories, Hercules, CA, USA) to determine the number of *SMN1* and *SMN2* gene copies. The ddPCR *SMN1* copy number kit (Bio-Rad Laboratories, Hercules, CA, USA) and ddPCR *SMN2* copy number kit (Bio-Rad Laboratories, Hercules, CA, USA) were used. A screen-positive result was defined as zero copies of *SMN1* exon 7 and four copies or less of *SMN2*. Carrier status and individuals with five or more *SMN2* copies were not reported.

Following a positive screening result, parents were contacted by a nurse from the PQDNS, given the positive screening results and invited to select one of three referral centers: (1) McGill University Health Centre—Montreal Children’s Hospital, (2) Centre Hospitalier Universitaire Sainte-Justine, or (3) Centre Hospitalier Universitaire de Québec—Université Laval [17]. The center was then promptly notified by the PQDNS, and an appointment was scheduled within two business days.

At the initial clinical visit, a complete neurological examination was performed and documented, and when possible, standardized motor assessments such as the Children’s Hospital of Philadelphia Infant Neurological Test for Neuromuscular Disorders (CHOP-INTEND) or the Alberta Infant Motor Scale (AIMS). Families received information about SMA and available treatment options. Confirmatory genetic testing and *SMN2* copy number determination were performed by Multiplex Ligation-dependent Probe Amplification (MLPA) on DNA extracted from a fresh blood sample. Anti-Adeno-associated virus (AAV9) antibody titers and basic laboratory work were performed concurrently. A standardized physiotherapy assessment was ideally performed prior to treatment initiation, without causing any delay in therapy.

Upon confirmation of diagnosis, a multidisciplinary management approach was initiated as clinically indicated, incorporating respirology (overnight pulse oximetry with or without chest X-ray for patients with respiratory symptoms), physiatry (for patients with contractures or scoliosis), dysphagia assessment (for those with bulbar symptoms or two *SMN2* copies), and genetics referral (for parental and cascade testing). Disease-modifying therapy was offered to patients with one to three *SMN2* copies, and to symptomatic patients with four *SMN2* copies. For asymptomatic patients with four *SMN2* copies, the decision to either immediately treat or conduct watchful waiting was made collaboratively with the family. If a patient was eligible for multiple therapies, the choice of treatment was guided by reimbursement criteria, anticipated delays in access, parental preference, and clinical judgment.

### 2.2. Data Collection

The clinical database for longitudinal follow-up was mandated by the Ministry of Health as a quality improvement initiative to oversee the well-functioning of the program and adjust the clinical pathway as needed. The database was maintained in REDCap at the McGill University Health Centre Research Institute (MUHC-RI). As a quality improvement database, a consent waiver was not required by the MUHC Research Ethics Board.

At the initial visit, demographic information and referral details were recorded. At the time of treatment initiation, results of genetic testing, including *SMN2* copy number, anti-AAV9 antibody titers, symptomatic status, and treatment details were documented. During each follow-up visit (every 6 months at a minimum), data were collected on growth parameters, motor function, bulbar and respiratory function (clinical variables such as dysphagia, feeding and ventilatory support), orthopedic issues, hospitalizations, changes in therapy, treatment-related adverse events, and mortality.

The following standardized assessments were used (or are planned for use) to monitor motor function, with the choice of test based on center preference: AIMS (≤18 months), World Health Organization Motor Milestones (≤2 years old), Hammersmith Infant Neurological Examination 2 (HINE-2) (2–24 months), CHOP-INTEND (≤2 years old or >2 years old if unable to sit independently), Bayley Scales of Infant Development IV full scale (BSID-IV) (at 24 months), and Hammersmith Functional Motor Scale Expanded (HFMSE) (≥2 years old).

### 2.3. Statistical Analysis

Statistical analyses were performed using IBM SPSS Statistics, Version 29.0. Categorical variables were summarized using frequencies and column percentages, while continuous variables were described by mean, median, and range. The 95% confidence interval (CI) for birth prevalence was calculated using the Wilson score method.

## 3. Results

In the first full year of the Quebec SMA NBS pilot program, 6 out of 67,933 tested newborns screened positive, all of whom subsequently had a confirmed diagnosis. Among these 6 newborns, 4 (67%) had two copies of the *SMN2* gene, and 2 (33%) had four copies (Table 1). All newborns with a positive screen were born at term. Although no false positives or false negatives were identified, one case outside the scope of the current screening algorithm was identified: a patient who initially screened negative with a single *SMN1* exon 7 copy but later developed symptoms. At the first neurology visit at 6 months of age, clinical examination revealed tongue fasciculations, severe hypotonia, severe generalized weakness predominantly affecting the lower extremities, gross motor delay (inability to sit or turn, poor head control), areflexia, and tachypnea with paradoxical breathing. Confirmatory genetic testing with sequencing revealed a compound heterozygous state, with a pathogenic variant (c.770_780dup p.(Gly261Leufs*8)) in the single *SMN1* copy. This patient, clinically classified as having SMA type 1B with two *SMN2* copies, received gene replacement therapy after a bridge with Risdiplam. Overall, the provincial birth prevalence of SMA in Quebec during the first year of the NBS program was 1 in 9705 live births (95% CI: 1 in 20,032 to 1 in 4701).

### 3.1. Affected Newborns with Two SMN2 Copies

Two of the four (50%) affected newborns with two *SMN2* copies showed no clinically evident signs at the time of their initial consultation, at the time of their infusion with onasemnogene abeparvovec, and at their 3-month follow-up. Case 1 had a CHOP-INTEND score of 46/64 at 28 days of life, which remained stable at 47/64 at 3 months. Case 2 was diagnosed prenatally with SMA due to a known affected sibling, allowing for expedited treatment initiation after birth by day of life nine. The initial clinical evaluation was performed on the first day of life, with both the newborn screening and confirmatory genetic testing conducted in parallel. This newborn’s CHOP-INTEND score increased from 54/64 at 9 days of life to 64/64 at the 6-month follow-up.

**Figure 1:**
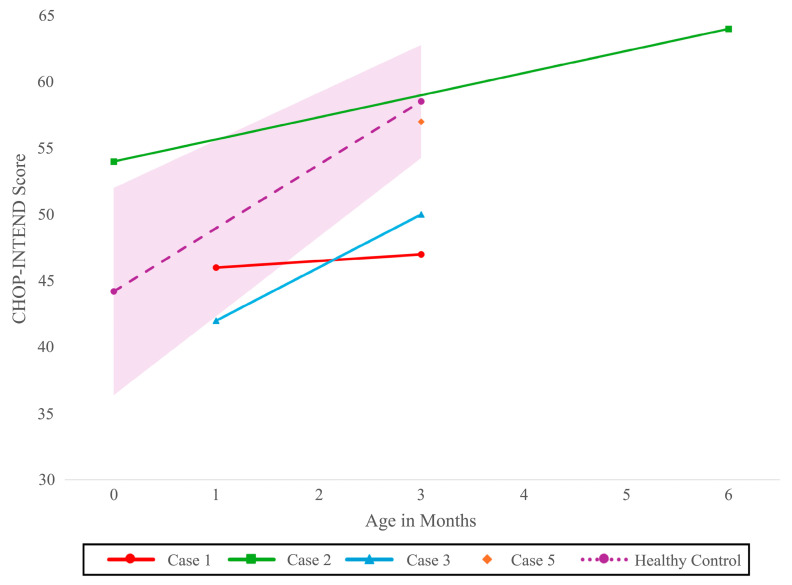
Course of CHOP-INTEND score over time for newborns with two *SMN2* copies (N = 3) and four *SMN2* copies (N = 1). Model based healthy control with 95% CI CHOP-INTEND [18]: Children’s Hospital of Philadelphia Infant Neurological Test Neuromuscular Disorders, *SMN2*: Survival motor neuron 2 gene.

One symptomatic newborn with two *SMN2* copies was already brought for evaluation when the result of NBS became available (Case 3). On initial consultation on day of life 16, clinical findings included tongue fasciculations, severe axial hypotonia, diffuse weakness of the lower extremities, proximal weakness of the upper extremities, and areflexia, accompanied by respiratory distress requiring admission to intensive care. Following multidisciplinary discussions involving the family and the neurology, respirology, social work, and palliative care teams, the infant was transitioned to comfort care and passed away at 43 days of life without receiving disease-modifying therapy. The fourth newborn with two copies of *SMN2* (Case 4) had early clinical signs at initial consultation and received onasemnogene abeparvovec at 30 days of life. Her CHOP-INTEND score improved from 42 at her initial visit (20 days of life) to 50 at the 3-month follow-up (Figure 1). At 3 months, no dysphagia, ventilatory support needs, orthopedic concerns, or hospitalizations were reported.

### 3.2. Affected Newborns with Four SMN2 Copies

Both newborns with four *SMN2* copies showed no clinically evident signs at the initial evaluation. Their AIMS scores remained stable and within normal limits between the initial evaluation and 3-month follow-up: Case 5 maintained a score of 13 (10th percentile), and Case 6 a score of 5 (50th percentile). Case 5 had a CHOP-INTEND score of 57/64 at the 3-month follow-up.

Case 5, who resided in a remote region of the province with limited access to specialized care, was treated with onasemnogene abeparvovec. He subsequently developed transient thrombocytopenia, a known adverse effect of the therapy [19]. He also developed silent aspiration on video fluoroscopy, requiring formula thickening at the 3-month follow-up; by 6 months, no clinical choking episodes were observed. Case 6 was treated with nusinersen and remained asymptomatic at 3 months with no reported adverse event.

### 3.3. Timeline of Diagnosis and Treatment Initiation

Newborns received a positive screening result at a median age of 17 days (range: 10–34 days), with initial clinical evaluations performed within three days of notification (median: 0 days) (Table 1). Confirmatory testing results were available by a median of 24 days of life (range: 3–41 days), and disease-modifying therapy was initiated at a median age of 30 days (range: 9–103 days).

Excluding the patient with a prenatal SMA diagnosis (Case 2) from the analyses had minimal impact on the median age at initial consultation (16.5 vs. 17 days) or confirmatory diagnosis (24 vs. 24.5 days); however, it increased the median age at treatment initiation from 30 to 37.5 days. Similarly, excluding the outlier (Case 5), who experienced the greatest delays in diagnosis and treatment, altered the median ages at key timepoints by no more than 0.5 to 1 day.

**Figure 2:**
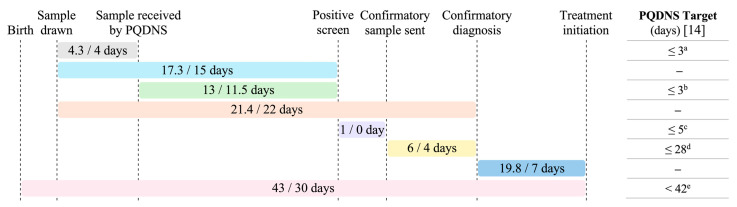
Number of days between key steps in the diagnostic and treatment process for newborns with SMA, relative to targets set by the PQDNS. Time intervals are reported as mean/median number of days. Of note, the confirmatory sample is typically collected at the time of the first clinic consultation; these two time points therefore coincide and are considered interchangeable. PQDNS: Programme québécois de dépistage néonatal sanguin (Québec Newborn Screening Program). ^a^ Target for ≥90% of samples, in business days. ^b^ Target for ≥99% of samples, in business days. ^c^ Target for 100% of newborns with a positive screen, in business days. ^d^ Target for 95% of newborns with SMA, in calendar days. ^e^ Target for 99% of newborns with SMA.

Figure 2 summarizes the time intervals between key diagnostic and treatment milestones, relative to targets set by the PQDNS. The longest interval occurred between the reception of the sample by the screening laboratory and the release of the positive screening result, with a median duration of 11.5 days (range: 6–24 days). This sample processing interval remained relatively stable over the study period and showed the greatest deviation from the PQDNS target of 3 days.

## 4. Discussion

This is the first report describing outcomes of the Quebec SMA NBS pilot program, which identified six newborns with SMA during its first year of progressive implementation. One infant with a compound heterozygous state (one *SMN1* copy with a pathogenic variant) was identified from symptoms; it is estimated that approximately 5% of newborns with SMA are compound heterozygotes and will not be detected by current NBS programs [2]. Although no false positives or false negatives were reported during the first year of SMA NBS programs in Quebec, Alberta and Ontario [13,14], such cases have been described in other countries and are expected to occur over time [20]. Based on these results, the estimated provincial birth prevalence of SMA in Quebec is 1 in 9705 live births, a rate consistent with those reported elsewhere in Canada (1:9401 in Alberta, [14] 1:27,960 in Ontario [13]) and internationally (1:6910 in Germany [21], 1:7890 in Spain [22], 1:11,471 in China [23], and 1:14,694 in the United States [24]). Assuming Hardy–Weinberg equilibrium, this corresponds to a carrier rate of 1:50.

The time intervals between key milestones for this program in Quebec were longer than in other Canadian provinces. In Quebec, screening samples were received by the laboratory at a median age of 6 days (range: 3–10 days), compared to 2 days in Alberta (range: 1–3 days) [14], and 3 days in Ontario (range: 3–6 days) [13]. These longer intervals may be partly explained by delays in sample transport, particularly for patients living farther from the screening laboratory, as well as reliance on regular postal services. Quebec spans 1.7 million square kilometers [25] and has a population of 9 million people [26], making it Canada’s largest province. Its vast territory poses significant challenges for the timely transport of screening samples. Notably, Case 5, who experienced the longest delays across all diagnostic and treatment milestones, resided in a remote region, thereby highlighting potential geographic disparities in access to diagnosis and care. However, even after sample receipt, the time to release positive screening results was longer in Quebec (median: 11.5 days) compared to Alberta (median: 4 days) [14] and Ontario (median: 6 days) [13], underscoring the need for improvements in sample testing and processing workflows. This was also the only step that deviated substantially from the targets set by the PQDNS. It is important to note that, in Quebec, both *SMN1* and *SMN2* copy numbers are determined and reported as part of the initial newborn screening, whereas in Alberta only *SMN1* is analyzed and reported. In contrast, the interval between initiating confirmatory diagnostic testing and obtaining results was relatively short in Quebec (4 days), comparable to Ontario (median: 4 days) [13] and faster than in Alberta (median: 8 days) [14]. Additional challenges, although not encountered during the program’s first year, must also be considered. For instance, in premature infants, NBS is often deferred until discontinuation of parenteral nutrition or hospital discharge, potentially delaying diagnosis and intervention.

For patients with SMA and four *SMN2* copies, a discrepancy exists between evolving consensus treatment recommendations and current public reimbursement criteria [27]. Because patients with four copies were excluded from pivotal disease-modifying therapy trials, early practice favored clinical monitoring with treatment initiated upon the emergence of symptoms [1]. However, growing natural history data for this subpopulation and evidence of phenotypic variability prompted revisions of consensus recommendations by experts, now favoring immediate treatment for all infants with four *SMN2* copies identified through NBS [28,29]. In Quebec, public reimbursement of onasemnogene abeparvovec is restricted to individuals with two or three *SMN2* copies, limiting publicly covered therapeutic options for newborns with four copies to nusinersen, as risdiplam is not yet recommended for reimbursement in infants < 2 months. Recognizing gaps in therapy access and the uncertainty surrounding outcomes in this subgroup, the Quebec SMA NBS algorithm incorporates a shared decision-making model for asymptomatic patients with four *SMN2* copies, allowing families and clinicians to decide collaboratively between early treatment or close clinical monitoring. Despite reimbursement constraints, both patients with four *SMN2* copies in our cohort elected to initiate disease-modifying therapy: one with nusinersen and one with onasemnogene abeparvovec. The rationale for selecting onasemnogene abeparvovec was the patient’s residence in a remote geographic region, which limited access to nusinersen and risdiplam. Improved alignment between clinical recommendations and reimbursement policies would facilitate more cohesive and equitable decision-making.

Our findings highlight key opportunities for optimizing the Quebec SMA NBS program. Priority should be given to strategies that reduce sample transport delays, especially for newborns from distant regions, and shorten the interval between sample receipt and reporting of positive screens. The PQDNS is updating its laboratory information system and automating analytical processes, which is expected to improve turnaround times. Standardization of motor function assessments across referral sites is also essential and should be informed by families’ experiences and feedback regarding the different evaluation tools. In addition, growing evidence of neurodevelopmental comorbidities in early-onset SMA, including developmental delays, autism spectrum disorder, and cognitive and language impairments [30,31], underscores the need to integrate neurodevelopmental outcomes into longitudinal follow-up.

## 5. Conclusions

While opportunities for optimization remain, the first-year experience of the Quebec SMA NBS program demonstrates the feasibility and success of identifying affected newborns early and facilitating timely access to disease-modifying therapies. Ongoing longitudinal follow-up will be crucial to understand the impact of early treatment on patients` clinical trajectories, inform future treatment choices, and support the evolving developmental needs of children with SMA.

## Figures and Tables

**Table 1:** Timeline and characteristics of patients with a positive newborn screen for SMA in Quebec (N = 6).

Steps	Case 1	Case 2 ^1^	Case 3 ^2^	Case 4	Case 5	Case 6	Mean	Median
	Days Since Birth							
Sample Drawn	3	2	1	1	2	1	1.7	1.5
Sample received by PQDNS	7	7	3	5	10	4	6	6
Initial positive screen	17	17	16	20	34	10	19	17
Parents contacted by selected referral site	17	N/A	16	20	34	11	19.6	17
First consult in clinic and confirmatory labs sent	17	0	16	20	37	12	17	16.5
Confirmatory diagnosis	24	3	N/A	23	41	25	23.2	24
First treatment initiation	28	9	N/A	30	103	45	43	30
**Patient Characteristics**								
Sex	F	F	M	F	M	M	-	-
Number of *SMN2* copies	2	2	2	2	4	4	-	-
Clinical signs at initial consultation	No	No	Yes	Yes	No	No	-	-
Treatment received	OA	OA	None	OA	OA	NUS	-	-

^1^ Prenatal diagnosis of SMA. ^2^ Redirection to palliative care and death at 43 days of life. F: Female, M: Male, NUS: Nusinersen, OA: Onasemnogene abeparvovec, PQDNS: Programme québécois de dépistage néonatal sanguin (Québec Newborn Screening Program), SMA: Spinal muscular atrophy, *SMN2*: Survival motor neuron 2 gene.

## Data Availability

The original contributions presented in this study are included in the article. Further inquiries can be directed to the corresponding author.

## Abbreviations

The following abbreviations are used in this manuscript:

AAV9	Anti-Adeno-associated virus
AIMS	Alberta Infant Motor Scale
BSID-IV	Bayley Scales of Infant Development IV
CHOP-INTEND	Children’s Hospital of Philadelphia Infant Neurological Test for Neuromuscular Disorders
HFMSE	Hammersmith Functional Motor Scale Expanded
HINE-2	Hammersmith Infant Neurological Examination part 2
INESSS	Institut national d’excellence en santé et en services sociaux (National Institute for Excellence in Health and Social Services)
NBS	Newborn screening
PCR	Polymerase chain reaction
PQDNS	Programme québécois de dépistage néonatal sanguin (Québec Newborn Screening Program)
SMA	Spinal Muscular Atrophy
*SMN1/2*	Survival motor neuron 1/2
